# Effects of Tomato Juice Intake on Salivary 8-Oxo-dG Levels as Oxidative Stress Biomarker after Extensive Physical Exercise

**DOI:** 10.1155/2020/8948723

**Published:** 2020-01-14

**Authors:** Ali Pour Khavari, Siamak Haghdoost

**Affiliations:** ^1^Centre for Radiation Protection Research, Dept. of Molecular Biosciences, the Wenner-Gren Institute, Stockholm University, Sweden; ^2^University of Caen Normandie, Cimap-Laria, Advanced Resource Center for HADrontherapy in Europe (ARCHADE), Caen, France

## Abstract

Reactive oxygen species (ROS) at a normal level are important molecules involved in several cellular processes including immune response and cell signalling. Overproduction of ROS may lead to elevated oxidative stress and consequently to age-related diseases. Most of the studies related to oxidative stress in humans have been done on blood samples. However, blood sampling might be painful, requires special qualified personnel, and has to be performed at medical centers. An alternative to blood is saliva. Saliva sampling is noninvasive and can be performed by the donor. Biomarker determination in saliva is becoming an important part of laboratory diagnosis, but method development is needed before it can be used in the clinics. In the present investigation, 16 donors performed extensive physical exercise by cycling and keeping their heart rate at 80% of maximum for 20 minutes. The physical activity was repeated 3 times: before tomato juice intake, after daily intake of 100 ml tomato juice during 3 weeks, and finally 3 weeks after finishing tomato juice intake (washout period). The level of the stress biomarker, salivary 8-oxo-dG, was determined before and after the physical activity. The results indicate that (a) 20 min extensive physical activity increases the level of 8-oxo-dG in saliva significantly (*p* = 0.0078) and (b) daily intake of 100 ml tomato juice may inhibit (*p* = 0.052) overproduction of salivary 8-oxo-dG by 20 min physical activity. We conclude that the 20 min extensive physical activity increases the level of salivary 8-oxo-dG in healthy donors and 100 ml daily intake of tomato juice may inhibit the increase of 8-oxo-dG in saliva.

## 1. Introduction

Different lifestyles such as smoking, physical exercise, and eating habits can either induce or reduce oxidative stress levels [1, 2]. Increased muscle activity has been linked to increasing levels of reactive oxygen species (ROS) due to elevated ATP production and oxygen consumption [2, 3]. It has been reported that elevated ROS, e.g., produced during extensive exercise or exposure to ionizing radiation, can cause damage to the biomolecules, while regular exercise results in adaptation of the body leading to resistance against oxidative stress through expression of antioxidant genes, e.g., superoxide dismutase and glutathione transferase [4]. Major endogenous cellular sources of ROS include mitochondria, NADPH oxidase, and xanthine oxidase [5].

Physiological levels of ROS are involved in normal cellular processes, e.g., apoptosis and immune response [6, 7] as well as production of normal muscle force [8]. However, during oxidative stress when the levels of ROS exceed the antioxidant capacity of cells, ROS may react with and modify the structures of proteins, lipids, deoxyribonucleotide triphosphates (dNTP), and DNA and disturb their physiological functions [9–11]. This may lead to muscle fatigue and contractile dysfunction [8] and initiate age-related diseases [12].

Among different ROS-induced modifications, DNA and dNTP modifications may lead to mutations. Different DNA modifications have been observed during exposure to ROS. Among DNA bases, guanine is most frequently subjected to oxidation due to its chemical structure [13–16]. One commonly studied guanine modification is 8-hydroxy-7,8-dihydro-2′-deoxyguanosine (8-oxo-dG) which has been used as a noninvasive biomarker for oxidative stress as it can be found in the extracellular fluids and ends up in urine, blood, and saliva [17–19]. In our previous studies, we have shown that the origin of extracellular 8-oxo-dG is the nucleotide pool where ROS react with dGTP molecules [15, 20]. We have set up a modified ELISA method for the detection of low concentrations of 8-oxo-dG in blood serum [15, 19, 21] and showed that tomato juice intake significantly reduces 8-oxo-dG increment in blood serum after extensive physical exercise [22] and protects cells from radiation-induced DNA damage [23].

However, in our previous studies, we used blood samples as a source of biomarkers. For blood collection, donors visited qualified medical personnel at medical centers.

The aim of the present project was to investigate whether salivary 8-oxo-dG can be used as an alternative to serum 8-oxo-dG and if the antioxidant effect of tomato juice intake could be observed by measuring 8-oxo-dG in saliva. In parallel, we wanted to determine the level of lycopene, one of the major antioxidants in tomato juice, in saliva to investigate its relation with salivary 8-oxo-dG concentration.

Saliva is a rich source of antioxidants, both enzymatic and nonenzymatic, that play a significant role for maintaining the redox balance in the oral cavity. It has been shown that the health status of the oral cavity is influenced by the levels of ROS [24]. The oral cavity is often exposed to ROS due to intake of alcohol [25], cigarette smoke [25, 26], medications, and diets rich in fat and protein [27, 28]. Another important source of ROS in the oral cavity is the presence of inflammation for elimination of pathogens, e.g., bacteria and fungi. During inflammation, ROS are produced by particular activated immune cells (monocytes and macrophages) to kill the pathogens. The ROS can damage the surrounding healthy tissues.

The following hypotheses have been tested: (1) extensive physical activity increases the level of 8-oxo-dG in saliva, (2) 100 ml daily intake of tomato juice for 3 weeks can inhibit production of 8-oxo-dG in saliva by extensive physical activity, and (3) there is a relationship between salivary 8-oxo-dG level and concentration of lycopene in saliva.

## 2. Material and Methods

The study was performed in accordance with the ethical standards and approved by the Swedish Ethical Committee at Karolinska University Hospital (dnr: 2018/59-32). For the study, 16 healthy individuals were recruited. The characteristics of the donors are presented in Table 1. Inclusion criteria were healthy individuals over age of 18 years. The exclusion criteria were the presence of autoimmune diseases, chronic or acute inflammation in the body [19, 29, 30], hypertension [31], diabetes [32, 33], and cancer [34–36]. All individuals were healthy, over 18 years old, nonvegetarian, and nonsmokers and did not take any vitamins or other food supplements 4 weeks prior to the investigation.

The participants were asked to have a daily intake of 100 ml tomato juice for 21 days followed by a washout period of 3 weeks without tomato juice intake. Saliva samples were collected at day 0 (E1), at day 21 (E2), and day 42 (E3, end of washout period). At the day for saliva sampling, the individuals were asked to perform 20 min of physical exercise using a stationary motion cycle (Monark Home Line 355). The heart rate was continuously monitored. The donors were informed to change the pedaling cadence to keep the heart rate constant at 80% of the maximum. To calculate the individual maximum heart rate, the following generally accepted formula was used: 220‐age = maximum heart rate. Two saliva samples were taken at each occasion, one before and the second 60 minutes after the exercise. Each participant was considered as their own control comparing the values before and after physical exercise. Prior to saliva sampling, the donors were asked to rinse the mouth 3 times with clean tap water. Saliva samples were collected in sterile tubes without any additive and kept at -20°C until analysis for lycopene and 8-oxo-dG contents. The level of 8-oxo-dG in saliva was analyzed as a marker of oxidative stress.

### 2.1. Measurement of Lycopene Concentration in the Tomato Juice

Lycopene concentration in the tomato juice was basically measured as described by Fish et al. [37]. The tomato juice was from the same manufacturer as in the previous publication [22]. Briefly, 1 ml acetone (NORMAPUR, VWR), 1 ml ethanol (96%, VWR) containing 0.1 mg/ml butylated hydroxytoluene (Sigma), and 2 ml hexane (Merck) were mixed in a glass tube with a Teflon-lined cap and kept on ice for 15 minutes. Thereafter, 0.1 ml of the tomato juice was added to the solution, shaken continuously for 30 minutes on ice, and kept in an ultrasonic bath for 7 minutes to extract lycopene. Then, 2 ml of cold ddH_2_O was added, the samples were mixed and centrifuged at 4000 × g for 5 minutes. The upper hexane layer was saved. The hexane layer was diluted 3 times, and the lycopene concentration was measured with a spectrophotometer at 503 nm in a 1 cm quartz cuvette with hexane as blank. The molar extinction coefficient 17.2 × 10^4^ M^−1^cm^−1^ was used for calculating the concentration of lycopene in hexane.

### 2.2. Measurement of Lycopene Concentration in Saliva

The lycopene content of saliva was determined essentially as described by Karppi et al. [38]. Briefly, 400 *μ*l of frozen saliva sample was thawed and mixed thoroughly with a solution containing 900 *μ*l cold ethanol containing 0.1 mg/ml BHT, 1600 *μ*l ddH_2_O, 100 *μ*l of 4 *μ*M apocarotenal in ethanol (Sigma) as internal standard, and 4 ml hexane. Following centrifugation, the solutions were kept at -80°C, and then, the hexane layer was decanted into a new tube. The tubes were kept on ice, and the hexane was evaporated to dryness under a stream of nitrogen gas. 200 *μ*l of ethanol containing 0.1 mg/ml BHT was added to each sample and mixed prior to HPLC analysis.

The samples were run through a 250 × 4.6 mm, C_30_, 5 *μ*m column (Stability®, Maisch) packed with porous spherical silica with a pore diameter of 100 Å and a surface area of 350 m2, and guard column 10 × 4.6 mm, Kromasil 100 C_18_, 5 *μ*m (Dalco Chromtech AB) with a flow rate of 1 ml/min with methanol (HPLC grade, Honeywell)/methylene chloride (analytical grad, Honeywell) (55 : 45 *v*/*v*) as liquid phase. Acquisition was made through a UV detector with a D2 lamp at 476 nm, and peaks were analyzed using the Clarity software version 7.3.0.3.

### 2.3. Purification and Determination of 8-Oxo-dG in Saliva

Efforts have already been done to measure 8-oxo-dG in saliva with controversial results [39–43] due to the presence of compounds in saliva that can influence the results; therefore, removing the compounds is an important step for quantitative biomarker detection particularly when an ELISA method is used. For this reason, we have used a basic protocol for the detection of 8-oxo-dG in blood serum and introduced several prepurification steps to remove the interfering compounds.

Briefly, frozen saliva samples were thawed at room temperature. 1.5 ml of the samples were transferred into new tubes and heated at 90°C for 5 minutes to reduce any enzymatic activity that may interfere with chemicals/antibodies used for the detection of 8-oxo-dG. The samples were cooled down to 4°C, then 10 *μ*l of 2.9 mM pepsin solution (Sigma-Aldrich) was added, and the samples were incubated at 37°C for 30 minutes to break down the mucosa to avoid clogging during filtration. Pepsin was deactivated by heating the samples to 90°C for 5 minutes. The samples were then centrifuged at 18000 × g for 30 min at 4°C, and the supernatants were transferred to Amicon Ultra 3 kDa 0.5 ml centrifugal filter devices (Merck) and centrifuged at 18000 × g for 60 minutes at 4°C. The filtrates were then applied on C18 solid phase columns according to the 8-oxo-dG protocol provided by Health Biomarkers Sweden AB [15, 20]. This step is necessary to remove products other than 8-oxo-dG which crossreact with the monoclonal antibody used in the kit.

A standard curve for 8-oxo-dG (0.01-10 ng/ml) was established for each plate covering the range of 8-oxo-dG concentrations in the samples. 270 *μ*l of each standard and sample was mixed with primary anti 8-oxo-dG antibody solution from the kit. The samples were then transferred to a 96-well ELISA plate that was coated with 8-oxo-dG and incubated at 4°C during night. The samples and the standards were loaded in triplicates. After incubation and washing, HRP-conjugated secondary antibody was added to each well and incubated for 2 hours at 24°C. The plate was washed, and the signals from the secondary antibody were determined by adding 140 *μ*l staining solution provided in the kit and incubating in the dark for 15-20 minutes at room temperature. The reaction was stopped by adding H_3_PO_4_ (Merck, Germany) solution, and the signal was read at 450 nm using an automatic microplate reader POLARstar Omega (BMG Labtech, Germany). A standard curve was established for each plate where the 8-oxo-dG concentrations of the saliva samples were determined. The validation of the modified ELISA method was previously performed by HPLC-EC (*r*^2^: 0.87, *p* < 0.05) [15]. Comparisons between the ELISA and the HPLC-EC methods showed a linear correlation at the concentration range found in human blood serum [15]. There was no correlation between ELISA and HPLC-EC when unfiltered samples were used.

### 2.4. Statistical Methods

The values are expressed as median and ranges due to skewed distribution of 8-oxo-dG data. Nonparametric Wilcoxon signed-rank test was used to test the statistical significance. A *p* value below 0.05 was deemed as significant.

## 3. Results

**Figure 1:**
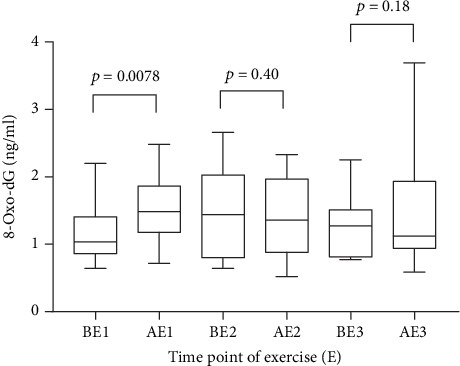
Saliva 8-oxo-dG (ng/ml) concentrations presented as median and ranges, before (B) and after (A) 20 minutes of physical activity: before tomato juice intake (E1), after 3 weeks daily intake of 100 ml tomato juice (E2), and after the washout period (E3).

At the beginning of the study, 20 minutes of exercise at 80% of maximum heart rate significantly increased (*p* = 0.0078) the 8-oxo-dG concentration in saliva (1.49 ranging from 0.72 to 2.48 ng/ml) as compared to the concentration before the exercise (1.04 ranging from 0.64 to 2.2 ng/ml) (Figure 1). After 3 weeks of intervention with a tomato juice intake of 100 ml/day (equal to 11 mg lycopene per day), no corresponding increase occurred and the concentrations before and after exercise were largely the same (1.44 ranging from 0.64 to 2.66 ng/ml and 1.36 ranging from 0.52 to 2.33 ng/ml, respectively).

**Figure 2:**
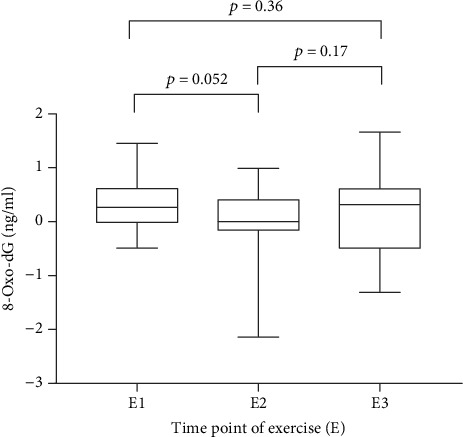
Represents increment of 8-oxo-dG as median and ranges where the 8-oxo-dG values before exercise have been subtracted from the values after 20 minutes of exercise. E1: 8-oxo-dG increment induced by physical activity; E2: 8-oxo-dG increment by physical activity after three weeks of juice intake; E3: 8-oxo-dG increment after the washout period.

Comparison of the increments due to cycling and tomato juice intake on the three analytical occasions (Figure 2, E1: 0.27 (-0.49 to 1.45), E2: 0 (-2.14 to 0.99), E3: 0.36 (-1.31 to 1.66)) shows a marginally significant effect (*p* = 0.052) of daily intake of tomato juice and an indication of a possible increase (*p* = 0.17) after the subsequent washout period.

To investigate the relation between 8-oxo-dG and lycopene, we tried to measure lycopene concentration in saliva and found that the level of lycopene in saliva was below the detection limit (approximately 10 ng/ml saliva) of the HPLC system used.

## 4. Discussion

The study demonstrates that 100 ml tomato juice intake, containing 11 mg lycopene, per day significantly protects the nucleotide pool from ROS in response to extensive physical activity. The explanation for the observed result is that the extensive physical activity requires ATP production which gives rise to the production of ROS. ROS react with dGTP and lead to the production of 8-oxo-dGTP in the cytoplasm [15, 20]. MutT homolog (MTH1) converts 8-oxo-dGTP into 8-oxo-dGMP [44, 45] to inhibit its incorporation into the DNA during DNA replication and repair [44]. 8-Oxo-dGMP is then converted to 8-oxo-dG by unknown enzyme(s) and excreted from the intra- to the extracellular milieu and enters all biological liquids including saliva. This mechanism has been in focus within our research group [15, 20, 45–47]. During the intervention after extensive physical activity, antioxidant content of tomato juice may react with ROS and keep their concentration low leading to lower production of 8-oxo-dGTP.

Analyses of 8-oxo-dG after a subsequent washout period of 3 weeks without tomato juice suggest that there may be an increase after exercise (Figure 2, E3). However, this increase was not statistically significant (*p* = 0.17).

It is important to mention that besides lycopene, tomatoes also contain other carotenoids, vitamin C, tocopherols, and polyphenols that may inhibit the reaction of ROS with dGTP and other biomolecules, and probably protect the cells from ROS-induced damage [19]. It has been shown that among all antioxidants (in particular carotenoids) present in tomato juice, lycopene and beta carotene are the most abundant and stable during industrial food processing [19] whereas most of vitamin C and tocopherols are destroyed by heating during food processing. Not much is known about the polyphenols in tomato juice [19]. In our previous study, we have determined lycopene and other carotenoids in blood serum of donors who took 3 weeks tomato juice and found that the combination of lycopene and other carotenoids has potential antioxidant properties that can reduce the level of DNA damage induced by ex vivo exposure of blood samples to gamma radiation [23].

The concentration of lycopene in blood plasma has been reported to be approximately 400 ng/ml [23, 48]. In the present study, we wanted to investigate whether it is possible to measure lycopene in saliva and, if possible, to investigate its relationship to salivary 8-oxo-dG. Ten saliva samples which were collected after 3 weeks of tomato juice intake were prepared for the analyses. However, we found that the level of lycopene in saliva was below the detection limit of the HPLC system used. The analytical method has previously been successfully applied to blood samples in our laboratory (data not shown).

The results of the current investigation support that the modified ELISA method and the saliva purification protocol are suitable for measuring 8-oxo-dG in saliva. An interesting finding in the present intervention study is that the level of 8-oxo-dG in human saliva was increased significantly after 20 minutes of acute physical activity possibly caused by an increase of the intracellular ROS level.

Further, we observed that a daily 100 ml tomato juice intake during 3 weeks may protect cells from the production of ROS after physical activity. The results are in accord with our previously published data [22].

The conclusions of the present investigation are the following: (a) extensive physical activity increases salivary 8-oxo-dG (*p* = 0.0078); (b) a daily intake of 100 ml tomato juice for 3 weeks seems to protect our body (*p* = 0.052) from the reaction of ROS, induced by the extensive physical activity, with dGTP and production of 8-oxo-dG in saliva; and (c) salivary 8-oxo-dG could be a candidate biomarker for oxidative stress levels in humans. However, considering the number of donors and the duration of the interventions (3 weeks), the obtained results should be considered as preliminary.

## Figures and Tables

**Table 1:** Characteristics of each donor. Degree of activity is given as None—no training, Low—training 1 day per week and out walking from time to time, Medium—training 2-3 times a week and other intense exercises, and High—athletic training, training 4-6 times a week, and intense muscle building work out.

Donor	Age	Gender	Degree of activity	Allergies
1	29	M	None	Yes
2	34	F	None	No
3	32	M	Medium	No
4	26	M	Medium	No
5	28	M	Low	No
6	34	M	Medium	No
7	29	F	None	Yes
8	41	M	High	No
9	25	F	Low	No
10	31	F	None	Yes
11	24	M	High	No
12	29	M	None	No
13	35	M	High	No
14	25	F	High	No
15	20	M	Low	No
16	32	M	Medium	No

## Data Availability

The data used to support the findings of this study are available from the corresponding author upon request.
